# Dynamic Softening Mechanism of Platinum Thermomechanically Deformed at Low Strain Rate

**DOI:** 10.3390/ma18040783

**Published:** 2025-02-11

**Authors:** Huiyi Tang, Baifeng Luan, Linjiang Chai, Fuen Zhang, Hongliang Liu, Yuchen Xiao, Mingyao Zhong, Baoan Wu

**Affiliations:** 1 State Key Laboratory of Mechanical Transmissions, Chongqing University, Chongqing 400044, China; hytang320@163.com (H.T.); zhangfuenallen@163.com (F.Z.); 2 Chongqing Materials Research Institute Co., Ltd., Chongqing 400707, China; xiaoyuchen@cmri.cc (Y.X.); zhongmingyao@163.com (M.Z.); 3 National Engineering Research Center for Instrument Functional Materials, Chongqing 400707, China; 4 College of Materials Science and Engineering, Chongqing University of Technology, Chongqing 400054, China; chailinjiang@cqut.edu.cn (L.C.); liuhongliang@stu.cqut.edu.cn (H.L.)

**Keywords:** pure Pt, low strain rate, thermal deformation, dynamic recovery, dynamic recrystallization

## Abstract

The mechanical behavior of pure Pt at elevated temperatures is critical for its high-temperature processing and applications. To understand its thermal deformation behavior and reach better processing control, thermal compression deformation was conducted for pure Pt in this work with a strain rate of 0.01 s^−1^ and temperatures ranging from 500 to 700 °C, followed by microstructure characterization by using electron backscatter diffraction (EBSD) technique. The results indicate that the grain size, fraction of low-angle grain boundaries, and dislocation density are generally reduced with increasing temperature. An analysis combining true stress–strain curves and microstructural characteristics indicates that dynamic recovery based on dislocation cross-slip/climb is always a main softening mechanism of pure Pt during thermal deformation. Continuous dynamic recrystallization and geometric dynamic recrystallization also occur when the deformation temperature exceeds 650 °C, which will effectively improve the microstructural homogeneity of pure Pt.

## 1. Introduction

Platinum (Pt) belongs to Group VIII of the Periodic table and is an important precious metal element. Pure Pt has a face-centered cubic (FCC) structure with a higher ductility than other known FCC metals like gold, silver, and copper [1,2]. Pure Pt and its alloys also have high melting points and high stacking fault energy (SFE) [3], equipping them with excellent plastic processing capabilities at high temperatures and enabling them to have extensive applications in aerospace, military, and defense industries (e.g., high-temperature sensors) [4]. In practice, pure Pt and its alloys are often subjected to various thermal processing, and the resultant microstructures and properties will be significantly influenced by the specific processing conditions [5,6]. To probe their thermal processing characteristics, researchers have attempted to conduct thermal compression testing for pure Pt and its alloys [7]. Thermal compression testing is an important technique for evaluating the response of materials under high-temperature and compressive loading. By adjusting processing parameters during testing, varied microstructures can be developed with different mechanical performances. The specific microstructural changes in pure Pt and its alloys during thermal compression are very complicated, highly relying on strain rates and temperatures.

Dynamic softening is one common phenomenon for metals subjected to the thermal deformation process, with their hardness and/or strength dropped with continuous deformation. The principal mechanisms of dynamic softening include dynamic recovery (DRV) and dynamic recrystallization (DRX) [8]. These two softening mechanisms can affect the flow behavior of a material through changing dislocation density, grain size, and crystallographic orientation [9,10]. More specifically, DRX contains several modes like continuous dynamic recrystallization (CDRX), discontinuous dynamic recrystallization (DDRX), and geometric dynamic recrystallization (GDRX) [11,12]. Earlier work demonstrates that thermal deformation at high temperatures and low strain rates suppresses DDRX but promotes CDRX behavior for metals with high stacking fault energy (SFE) [13]. Li et al. [14] found that the high SFE of an Al-Mg-Si alloy facilitated the development of low-angle grain boundaries (LAGBs) in the initial stage of thermal deformation, leading to the predominance of DRV. As deformation progresses, with the continuous absorption of dislocations and subgrain rotation, LAGBs will be gradually transformed into high-angle grain boundaries (HAGBs), leading to the formation of small grains with serrated boundaries. When these serrated grains come into contact with each other, a “pinch-off” phenomenon may occur, and many new grains are then produced (known as GDRX) [15]. Currently, although a consensus on the GDRX mechanism is not reached, its distinct difference from the CDRX is widely recognized [16,17]. Zhang et al. [18] performed thermal deformation for a Pt-10Ir alloy at high temperatures and low strain rates and found that its grain structures tend to be squashed with deformation, along with active behaviors of both CDRX and GDRX. Earlier work on Pt-based materials is mainly focused on Pt alloys with very little attention paid to pure Pt, and the understanding of the thermal softening mechanism of pure Pt is still very limited. Studying its softening mechanism of thermal deformation at low strain rates allows for a better understanding of dynamic recrystallization and recovery behavior of the material, which has a significant impact on grain size, dislocation density, and mechanical performance of the material. By clarifying microstructural changes in pure Pt, it allows us to predict its deformation behavior under different processing conditions and reach better processing control.

In this work, therefore, pure Pt rods were subjected to thermal compression at a low strain rate (0.01 s^−1^) and various temperatures (500–700 °C). Detailed microstructural characterization was carried out for the specimens before and after the thermal deformation by mainly using electron backscatter diffraction (EBSD). The effect of processing temperatures on the microstructure was studied with dynamic softening mechanisms analyzed carefully. This work should be able to provide an insightful reference for realizing the thermal processing characteristics of pure Pt and its high-temperature application potential.

## 2. Experimental

**Figure 1:**
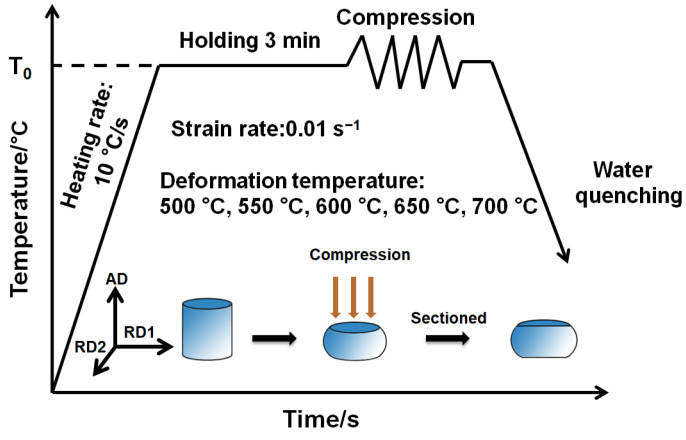
Schematic of thermal compression experiment (AD corresponds to axial direction of specimen, while RD1 and RD2 represent its two orthogonal radial directions).

The as-received material is a pure Pt (purity ≥ 99.9% with melting temperature of 1769 °C) rod (8 mm in diameter) prepared through thermal drawing. Utilizing a wire-cutting device, cylindrical specimens, with the size of 8 mm in diameter and 10 mm in height, were sectioned from the rod for thermal compression testing. Before the thermal compression, tantalum foils were placed between the pressure head and the specimens, with their top and bottom surfaces pasted with graphite to prevent adhesion at elevated temperatures and reduce the effect of friction. These specimens were subjected to thermal compression testing using a Gleeble-3800 thermal compression simulator (DSI, Albany, NY, USA) at five temperatures (500, 550, 600, 650, and 700 °C), with a fixed strain rate of 0.01 s^−1^. The thermal compression simulator was set at a heating rate of 10 °C/s and kept 3 min after reaching the set temperature. The isothermal compression experiment was terminated when the compression amount reached 60% (corresponding to a true strain of 0.9), followed by rapid quenching to retain the high-temperature microstructure, as schematically illustrated in Figure 1.

The specimens compressed at different temperatures were halved along the compression direction, with one half of them selected for the following study. Each specimen was successively polished using 180–1200 # SiC sandpapers and washed with anhydrous ethanol to remove surface contaminants, followed by electropolishing using a mixture of 40 mL saturated NaCl and 10 mL HCl as the electrolyte (temperature 25 °C, alternating voltage 11 V and duration 60 s). The electropolished surfaces were further processed using an argon ion polishing machine (Fischione 1061, Fischione Instruments, Pittsburgh, PA, USA) to obtain an even flatter surface (6 kV for 30 min and 4 kV for 15 min). Microstructures of the specimens were then characterized using a field emission scanning electron microscope (Thermal Fisher Scientific Apreo 2s, Waltham, MA, USA), with the EBSD data collected by the NordlysMax^2^ (Oxford Instruments, Abingdon, UK) detector with AZtec 5.1 software (step size of 4 μm), followed by data post-processing using AZtecCrystal 2.1 (grain measurement standard: ASTM E2627–2013; method: equivalent circle diameter; number of fields of view: 1; sample: 1) (Oxford Instruments, Abingdon, UK) and HKL Channel 5 software (version: 5.0.9.1).

## 3. Results

### 3.1. Initial Microstructure

**Figure 2:**
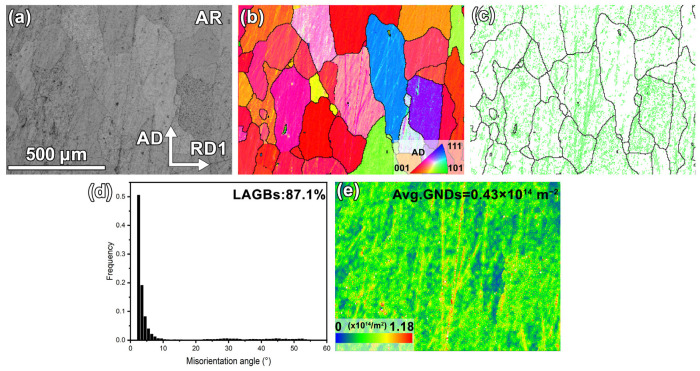
EBSD characterization results of as-received specimen: (**a**) BC map, (**b**) IPF map, (**c**) GB map, (**d**) MAD histogram, and (**e**) GND distribution map.

The EBSD characterization of the as-received specimen is presented in Figure 2, where Figure 2a (band contrast (BC) map) shows that its microstructure primarily consists of coarse blocky grains. The inverse pole figure (IPF) map (Figure 2b) reveals scattered grain orientations, with most grains having their long axes aligned toward AD. By using the “equivalent circle diameter” method [19], its average grain size is determined to be 121.9 μm. Figure 2c displays the grain boundary (GB) map with HAGBs (θ > 15°) and LAGBs (2.5° < θ < 15°), indicated by black and green lines, respectively, from which numerous LAGBs are found to exist in most grains. The misorientation angle distribution (MAD) map (Figure 2d) further indicates that this specimen has a LAGB fraction of 87.1% in. Figure 2e is the distribution map of geometrically necessary dislocations (GND), which clearly reveals that there are high-density dislocations in most grains. The above features indicate that the as-received pure Pt has typically deformed microstructures.

### 3.2. Thermocompression Flow Behavior

**Figure 3:**
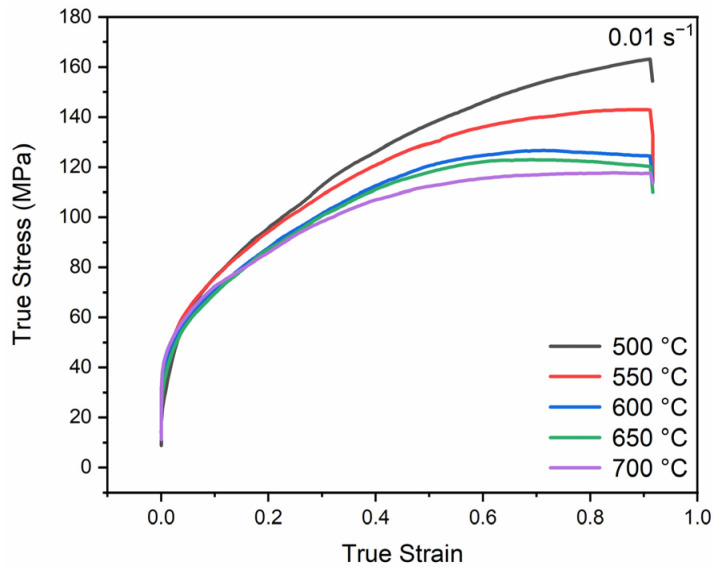
True stress–strain curves of specimens subjected to thermal compression at various temperatures.

The true stress–strain curves of pure Pt compressed at 500–700 °C are shown in Figure 3 and the flow stress is found to always decrease as temperature rises, suggesting a significant influence of temperature on its flow behavior. In general, the true stress–strain curves of all specimens can be divided into two stages: an initial work hardening stage (ε ≤ 0.2) and a subsequent relatively stable stage (0.2 < ε ≤ 0.9). In the initial stage, the true stress rises steeply, which is associated with the rapid increase in dislocation density. Dislocations propagated during the thermal deformation will become entangled with each other and limit their movement, leading to significant work hardening [20]. Following the work hardening stage, as strain further increases, the DRV begins to work and will cause a certain degree of strain softening [21]. The underlying mechanism behind this phenomenon is that under the conditions of high-temperature and low strain rates, the dislocation density could be gradually reduced through dislocation annihilation and/or re-alignment to become subgrain boundaries. This will not only alleviate the internal stresses due to dislocation pile-ups but also mitigate the work hardening effect to some extent. With increasing temperature, the rising trend of its curve gradually slows down and the corresponding flow stress decreases, which may be related to DRX [22].

### 3.3. Microstructural Characteristics After Thermal Deformation

**Figure 4:**
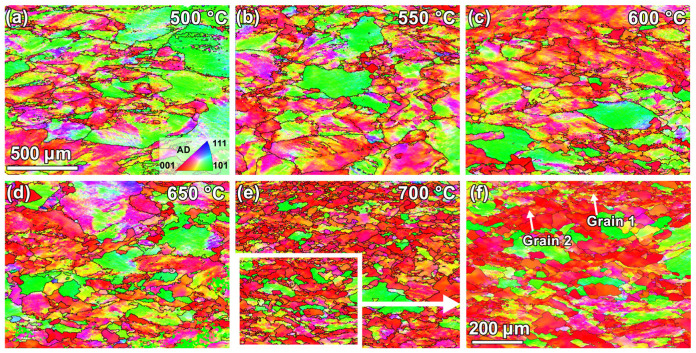
IPF maps of specimens subjected to thermal compression at various temperatures: (**a**–**e**) 500–700 °C; (**f**) boxed region in (**e**).

**Figure 5:**
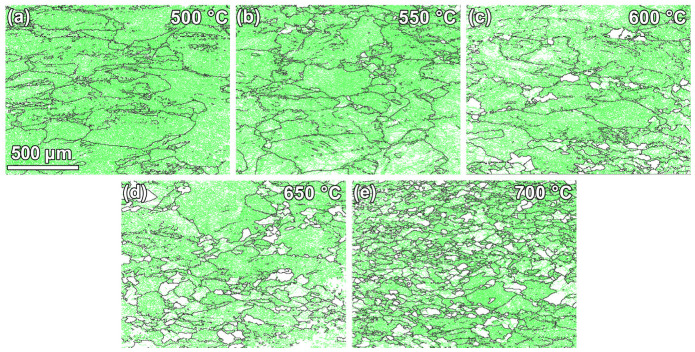
GB maps of specimens subjected to thermal compression at various temperatures: (**a**–**e**) 500–700 °C.

The specimens compressed at different temperatures are shown in the IPF and GB maps (Figure 4 and Figure 5). Comparing Figure 2b and Figure 4 reveals that at lower temperatures (600 °C and below), most grains tend to have their long axes along AD in the as-received specimen tilted toward RD1. This change should be related to the continuous compression of grains along the loading direction (AD) during the thermal deformation. Table 1 indicates that the grain sizes of these specimens are generally reduced after thermal deformation, compared to that of the as-received specimen. When the deformation temperature exceeds 650 °C, very significant grain refinement can be noticed, which may be related to the occurrence of DRX. Meanwhile, microstructure homogeneity seems to also be gradually improved, as indicated by the reduced discrete degree of grain sizes.

**Figure 6:**
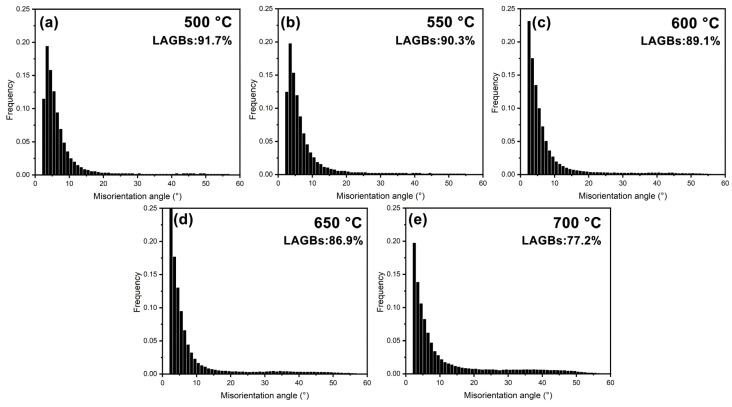
MAD histograms of specimens subjected to thermal compression at various temperatures: (**a**–**e**) 500–700 °C.

The MAD histograms of these thermally compressed specimens are displayed in Figure 6. From Figure 2d and Figure 6, the misorientation angles of all specimens in this study are mainly concentrated below 20°. Among them, the fractions of LAGBs in the specimens deformed at 500–600 °C are near 90%, slightly higher than those of the as-received specimen (87.1%), which should be related to the predominance of DRV at this stage. As the deformation temperature increases (up to 700 °C), the fraction of LAGBs gradually decreases, suggesting that DRX become active and promoted the transformation of LAGBs to HAGBs.

**Figure 7:**
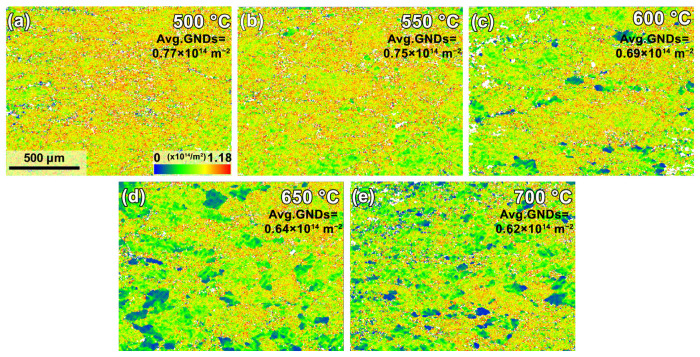
GND distribution maps of specimens subjected to thermal compression at different temperatures: (**a**–**e**) 500–700 °C.

Geometrically necessary dislocations are dislocations generated to coordinate the lattice deformation and strain and are also regarded as an important indicator for plastic strain and work hardening behavior. A higher GND density usually indicates more significant plastic deformation. Figure 7 shows that all thermally compressed specimens have higher GND densities than those of the as-received specimen (0.43 × 10^14^ m^−2^ in Figure 2e). This indicates that considerable propagation of GND has occurred during thermal compression. The GND density of the 500 °C specimen is almost twice that of the as-received specimen. Raising the deformation temperature can provide more external energy, allowing more significant DRV/DRX to occur and leading to decreased GND densities.

## 4. Discussion

During the thermal deformation of pure Pt, dense dislocations will first be generated with increasing applied stress, leading to work hardening. Since the deformation is performed at elevated temperatures, DRV and DRX may gradually occur to suppress work hardening. The DRX mechanism of high SFE metals (including pure Pt) usually prefers CDRX during the thermal deformation [13]. In this study, however, many serrated grain boundaries occur in the compressed specimens at high temperatures, which is consistent with the main characteristics of GDRX.

**Figure 8:**
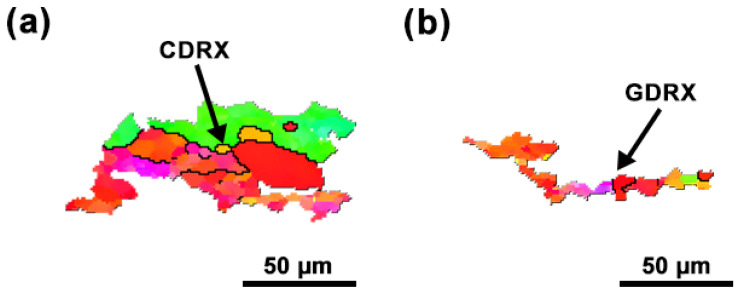
Typical structures extracted from Figure 4f: (**a**) CDRX nucleation and (**b**) GDRX nucleation. The color code is the same as that in Figure 4.

For further probing the DRX characteristics of pure Pt, typical microstructures in Figure 4f (the 700 °C specimen) are extracted and analyzed, as shown in Figure 8. Figure 8a illustrates that new grains (with sizes ~10–20 μm) mainly appear at the original grain boundaries of the specimen, corresponding to a typical feature of CDRX for high SFE metals. The preferred occurrence of CDRX in high SFE materials is mainly due to their easy dislocation annihilation through cross-slip and climb, promoting the formation of subgrains. With accumulating more strains, misorientation angles of these subgrain boundaries continuously increase by absorbing dislocations, and recrystallization nuclei are then formed by subgrain rotation and/or coalescence [23,24]. From Figure 8b, it can also be observed that the grains are markedly elongated and narrowed in specific regions, with serration features appearing at their boundaries. These serrated grain boundaries gradually come into contact with each other under continuous thermal loading. When the internal dislocation network approaches the grain boundaries, the “pinch-off” phenomenon will occur at these boundaries due to the effect of surface tension, leading to the formation of new equiaxed grains (the occurrence of GDRX).

**Figure 9:**
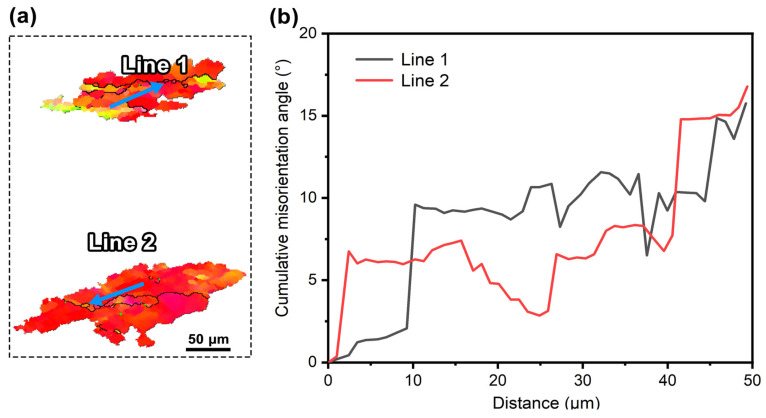
(**a**) Morphologies of two typical deformed grains (Grain 1 and Grain 2) extracted from Figure 4f (as indicated by white arrows in Figure 4f); (**b**) cumulative misorientations angle along Line 1 and Line 2 in (**a**). The color code in (**a**) is the same as that in Figure 4.

The measurements of cumulative misorientation angles inside two typical deformed grains in Figure 4f are presented in Figure 9. One can see that from the grain interiors to boundaries, their cumulative misorientation angles continuously increase and could exceed 15° at some distances. This indicates that there exists significant lattice rotation in the thermally compressed pure Pt, making necessary preparation for the occurrence of subsequent CDRX [24,25].

**Figure 10:**
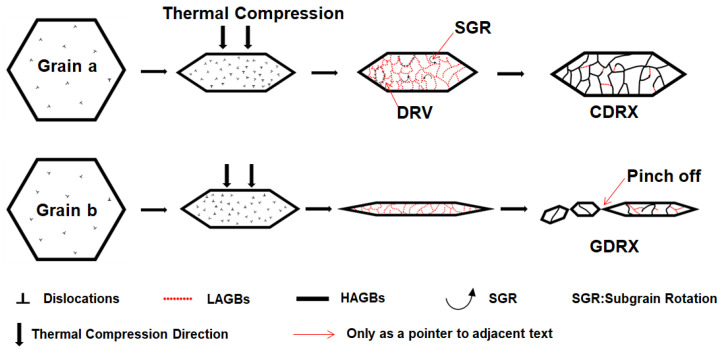
DRX mechanisms of pure Pt compressed at elevated temperatures.

The mechanism schematic of DRX behaviors of pure Pt subjected to thermal compression in this study is presented in Figure 10. In the figure, Grains a and b represent typical initial grains (with relatively low dislocation densities). After thermal deformation is initiated, their grain sizes gradually decrease and is accompanied by a rapid increase in dislocation density. As thermal deformation progresses, newly generated dislocations continuously undergo cross-slip and climb, leading a large number of subgrains to be formed through DRV. When the accumulated strain is relatively large, subgrain rotation (SGR) happens under external stresses. Then, the subgrains surrounded by LAGBs are gradually transformed into recrystallization nuclei enclosed by HAGBs, i.e., the occurrence of CDRX [26]. Meanwhile, during thermal deformation, some grains are considerably elongated due to external stresses. When the sizes of the subgrains inside them are reduced to a certain value, the “pinch-off” phenomenon occurs at the subgrain boundaries, thus generating new recrystallization nuclei, i.e., the occurrence of GDRX. Both the dynamic recrystallization behaviors could help improve the microstructure homogeneity and facilitate subsequent processing [27].

## 5. Conclusions

In this work, the study of the softening mechanism of pure Pt thermally deformed at low strain rates provides a theoretical basis for optimizing thermal processing and fills the gap in the study of the softening mechanism of pure Pt. A limitation of this work is that only a single strain rate was used, and we will consider expanding a wider range of temperatures and strain rates in our future work to more comprehensively understand the thermal deformation behavior of pure Pt. The main conclusions of this study are as follows:

1. During thermal compression deformation at 0.01 s^−1^, the grain size, LAGBs fractions, and dislocation density of pure Pt generally decrease with increasing temperature.
2. DRV based on dislocation cross-slip/climb is always found as a softening mechanism for pure Pt compressed at 500–700 °C.
3. After the deformation temperature exceeds 650 °C, both CDRX and GDRX occur, facilitating improving the microstructural homogeneity.

## Figures and Tables

**Table 1:** Average grain sizes of different specimens (μm).

As-Received	500 °C	550 °C	600 °C	650 °C	700 °C
121.9	60.1	59.8	61.8	57.3	43.4

## Data Availability

The raw/processed data required to reproduce these findings cannot be shared at this time as the data also form part of an ongoing study.
